# Acute pancreatitis after thoracic duct ligation for iatrogenic chylothorax. A case report

**DOI:** 10.1186/s12893-017-0204-3

**Published:** 2017-01-23

**Authors:** Benoît Bédat, Cosimo Riccardo Scarpa, Samira Mercedes Sadowski, Frédéric Triponez, Wolfram Karenovics

**Affiliations:** Thoracic and Endocrine Surgery, University Hospitals of Geneva, 1211 Geneva, Switzerland

**Keywords:** Case report, Thoracic surgery, Thoracic duct ligation, Acute pancreatitis, Iatrogenic chylothorax, Thymoma

## Abstract

**Background:**

To report the association between thoracic duct ligation and acute pancreatitis. The association between sudden stop of lymphatic flow and pancreatitis has been established in experimental models.

**Case presentation:**

*A* 57-year-old woman operated for thymoma presented a iatrogenic chylothorax. After thoracic duct ligation, she presented an acute pancreatitis which resolved after conservative treatment. The chylothorax disappeared within 4 days of thoracic duct ligation.

**Conclusions:**

This is the first report of acute pancreatitis following thoracic duct ligation. The pancreas and digestive tract should be assessed in symptomatic patients after thoracic duct ligation.

**Electronic supplementary material:**

The online version of this article (doi:10.1186/s12893-017-0204-3) contains supplementary material, which is available to authorized users.

## Background

Chylothorax is a rare disease defined as a leakage of chyle into the pleural space, and can be classified as traumatic or non traumatic. Esophageal surgery is the major cause of traumatic iatrogenic chylothorax. Other iatrogenic causes include lymph node dissection, lung resection and mediastinal tumor resection [1, 2]. A large leak flow of chyle may cause dehydration, nutrient loss and immunodeficiency. However, there is no consensus in the management of chylothorax. Depending on etiology, duration and flow output, therapy may either be conservative or surgical. Low-output chyle flow (<1000 mL/day) can generally be managed conservatively. Failure of conservative treatment on the other hand, typically in situations of high-output chyle flow (>1000 mL/day), requires intervention, such as thoracic duct ligation. This procedure has a high success rate of up to 95% [3]. Thoracic duct ligation has shown to have a 38% rate of comorbidities linked to procedure, such as atrial fibrillation and need of prolonged ventilation [2]. To our knowledge, there have been no abdominal complications reported or associated with ligation of the thoracic duct. In experimental models, some studies demonstrated that the sudden stop of lymphatic flow may induce intestinal and pancreatic edema with the presence of an inflammatory infiltrate [4, 5].

We report herein the first case of an abdominal complication associated with thoracic duct ligation, such as acute pancreatitis.

## Case presentation

**Fig. 1:**
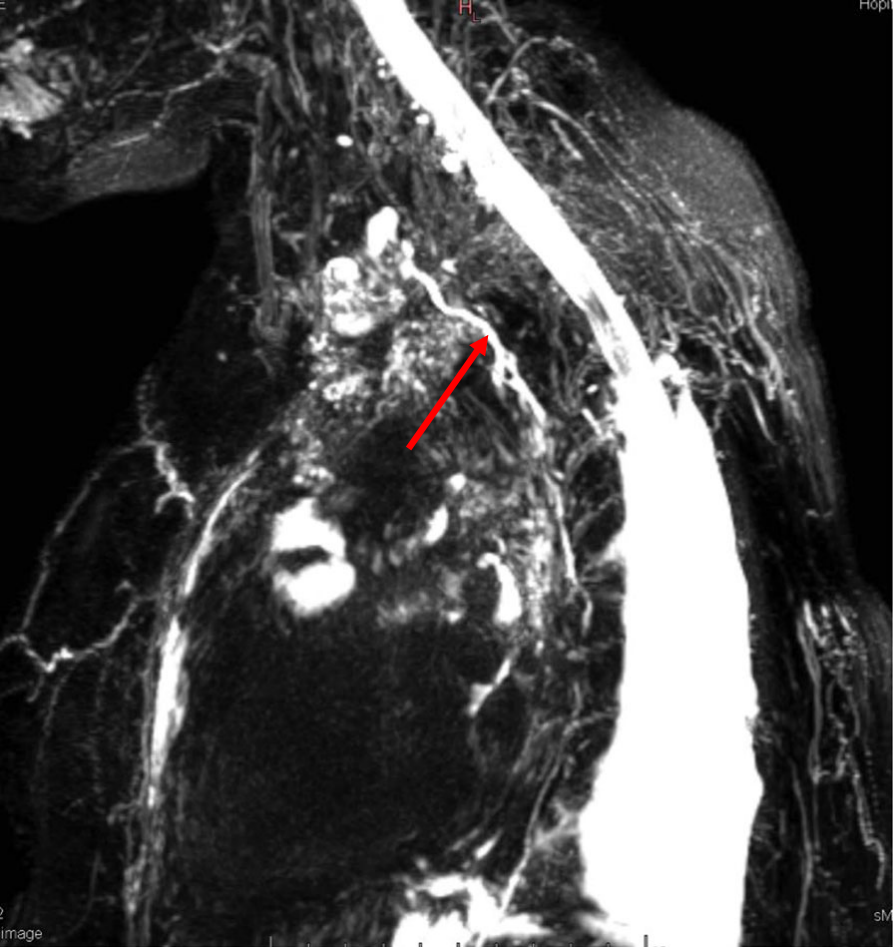
Axial MRI T2 showing the thoracic duct (*red arrow*)

**Fig. 2:**
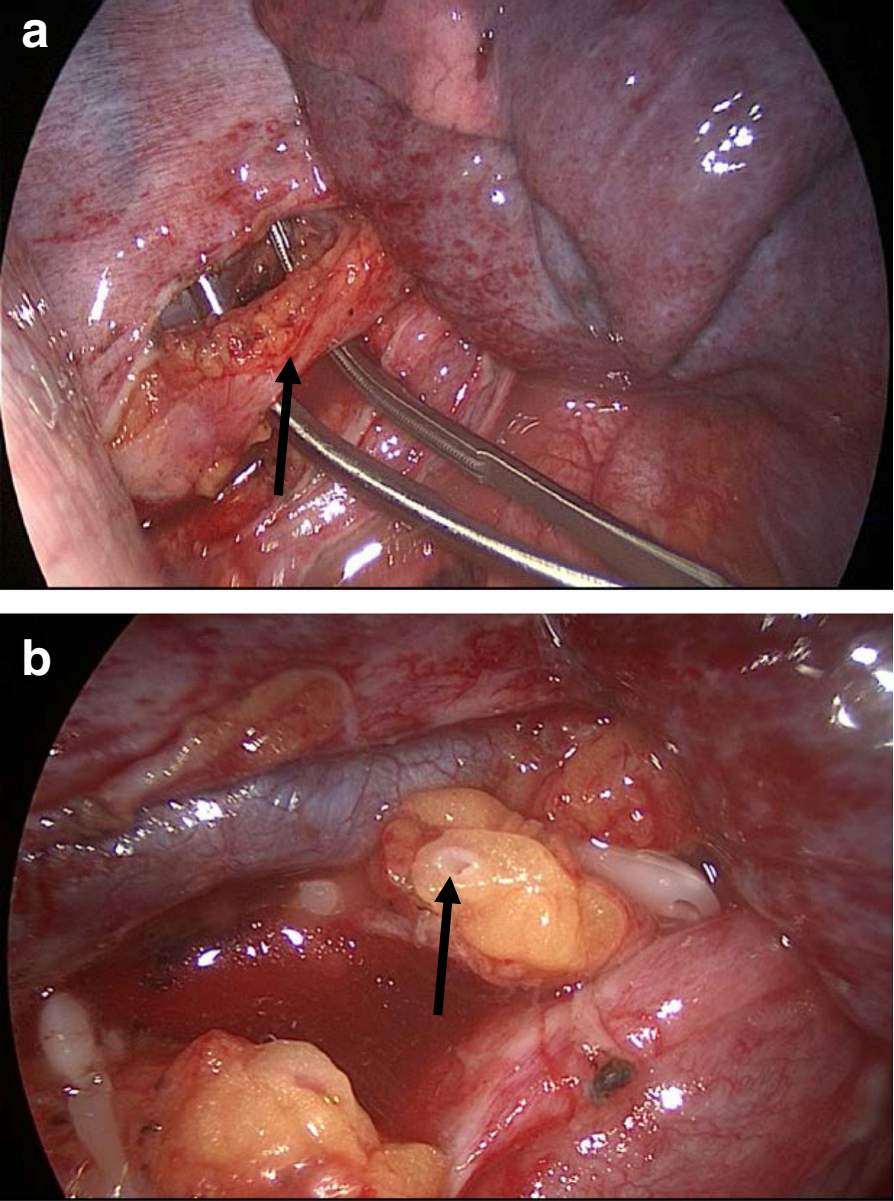
Dissection (**a**), clipping and section (**b**) of the thoracic duct (*black arrow*)

**Fig. 3:**
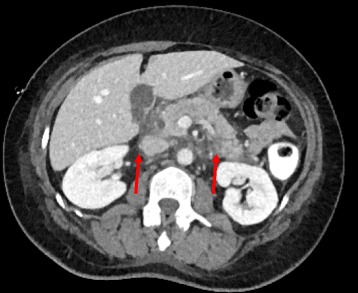
Arterial phase contrast CT showing an acute pancreatitis with edema around the pancreas (*red arrows*)

A 57-year-old woman with a history of back pain underwent a thoracic CT-scan in 2016 with the incidental discovery of an anterior mediastinal mass. The mass had a size of 4x3cm, was round and was well delimited, compatible with a teratoma or a thymoma. The patient had no symptom or clinical manifestations of myasthenia gravis, with a Quantitative Myasthenic Gravis Score grade 0. A thymomectomy was performed using a right single-port video-assisted thoracoscopic surgery (VATS). A chest drain was left after the procedure. Histopathology confirmed a thymoma, type B1, Masaoka-Koga stage 1. Two days after surgery, the patient developed a high output chylothorax (>1000 mL/day), without any signs of infection or inflammation in the blood tests. We introduced a low-fat diet for 6 days and then she was fasting for two more days. However, the chyle continued to flow with an output of 750 mL/day without other complications. No somatostatin or analog was introduced. A lymphangio-magnetic resonance imaging (MRI) didn’t show aberrant thoracic duct anatomy nor chyle-leak (Fig. 1). At day 10, the patient was taken to the operating room for a right VATS. The thoracic duct was visualized and the thoracic duct was clipped just above the diaphragm (Fig. 2). Two days later, the chylothorax reappeared and the patient developed increasing pain in the left hemi-abdomen, with sign of peritonitis and abdominal distention. Her blood test showed absence of leucocytosis, a C-reactive protein (CRP) of 230 mg/L, and normal lipid, electrolytes, hepatic and pancreatic levels (see Additional file 1). A CT-scan demonstrated pancreatic edema and a peri-pancreatic infiltration that extended to the bilateral kidney fasciae, compatible with an acute pancreatitis (Fig. 3). Etiologies of acute pancreatitis such as gallstone migration, alcohol, medications, hypotension during the perioperative period, lipidic and IgG4 disease were excluded (see Additional file 1). There was no hypotension during the perioperative periode. After medical supportive management the abdominal pain resolved within 2 days and CRP decreased. The chylothorax was treated with restricted low-fat diet, resolved 4 days after the thoracic duct ligation, and the chest tube was removed 16 days after the initial thymomectomy. Six months later, the patient is healthy and has had no recurrence of chylothorax.

This case presentation was conducted in accordance with the CARE guidelines and methodology.

## Conclusions

The occurrence of chylothorax after thymomectomy is a rare complication and usually associated with extensive dissection [6]. In this case, the resection was localized, without radical thymectomy.

For chyle leak of traumatic iatrogenic etiology, such as mediastinal tumor resection, management is debated and to date no consensus exists. Initial conservative treatment involving chest drainage and a low-fat diet except for the medium-chain triglycerides or fasting with parenteral nutrition has shown excellent outcome. Octreotide or somatostatin therapy can be efficient for the medical approach [7]. However, the level of evidence about the efficacy of these treatments remains low. Furthermore, octreotide is a class II medication associated with acute pancreatitis, and its use should be cautious [8]. For patients who fail conservative management, surgical repair with ligation using VATS is shown to be highly successful and is considered a safe procedure [3]. The difficulty lies in the visualization of the thoracic duct injury during the surgery and its anatomic variations. A lymphangiography, lymphoscintigraphy and lymphangio-MRI may help localize the chyle-leak. In this case, the leak site was not found on lympangio-MRI nor during surgery. An intraoperatively administration of oral cream via a stomach probe can be more easily identify the duct and other accessory lymphatic channels [9]. In this situation, the upstream clipping of the thoracic duct just above the diaphragm appears favorable for better result than a mass ligation of the tissue in the presumed course.

The consequences of sudden disturbed lymph drainage of the abdominal viscera, which constitutes 80% of the lymphatic flow in the thoracic duct, are not known. It is assumed that with time collateral lymphatic vessels overcome the thoracic duct obstruction. Reports in literature show that thoracic duct ligation is safe and has no known associated abdominal or lymphatic complications, except two cases of leg edema associated with thoracic duct embolization [10]. However, the role of mesenteric lymph drainage in acute illness such as pancreatitis, burns and hemorrhagic shock is well established in experimental models [11]. The impact of thoracic duct ligation on the pancreas is known since 1958 by Papp et al. and was more investigated by Müller et al. in 1988 with thoracic duct ligation in rats [4, 12]. Their results demonstrated a long-lasting pancreatic edema. A more recent study showed that thoracic duct ligation in rats with acute hemorrhagic necrotizing pancreatitis reduced lung injury by a decreased neutrophil infiltration and TNF-alpha release, but increased pancreas injury [13]. In the intestine and the pancreas, the myeloperoxidase activity, a marker of neutrophils infiltration, was increased without any change of serum amylase and diamine oxidase level. In another study, lymphatic obstruction in dogs caused an intestinal mucosal atrophy similar to malabsorption syndrome [14].

In our case, common etiologies of acute pancreatitis were excluded, including hypotension and medications. Auto-immune-like pancreatitis was previously described in a patient with myasthenia gravis and autoantibodies [15]. However, our patient had no symptom or clinical manifestations of myasthenia gravis with a Quantitative Myasthenic Gravis Score grade 0. Therefore an immune etiology seemed unlikely. The diagnosis of pancreatitis is only based on the CT-scan. Otherwise, acute pancreatitis with normoamylasemia and lipasemia is not uncommon, and is a known entity [16]. Another explanation could be a pancreatic edema caused by a congestive lymphatic vessels with similar symptoms. The comparison of the experimental studies with our case is clearly limited by their experimental design. However, the impact of a sudden lymphatic obstruction on the pancreas and digestive tract can be easily understood.

The recurrence of chylothorax 2 days after surgery evokes aberrant collateral thoracic duct, which has not been seen on MRI nor during surgery. Probably this duct was small with a low chyle flow that could explain a rapid healing after a conservative treatment and a persistant disturbed lymph drainage of the abdominal viscera with abdominal pain.

In conclusion, this is the first report of a patient developing acute pancreatitis after thoracic duct clipping. Although our knowledge relies on experimental models, edema of the abdominal viscera should be assessed in symptomatic patients with a CT-scan and a pancreatic enzymes analysis.

## Additional file

Additional file 1: Description of data : Blood test results 1 day after the onset of the abdominal pain. WBC, white blood count; CRP, C-reactive protein; AST, aspartate aminotransferase; ALT, alanine aminotransferase; γGT, gamma-glutamyl transpeptidase; AP, alkaline phosphatase; IgG, immunoglobulin G. (DOCX 14 kb)

## Abbreviations

CRP: C-reactive protein
MRI: Magnetic resonance imaging
VATS: Video-assisted thoracoscopic surgery
