# Supplementary material for: Acute pancreatitis after thoracic duct ligation for iatrogenic chylothorax. A case report
Source: BMC Surg. 2017 Jan 23;17:9. doi: 10.1186/s12893-017-0204-3 (PMC5260084; doi:10.1186/s12893-017-0204-3)
Supplement: Additional file 1: — Description of data : Blood test results 1 day after the onset of the abdominal pain. WBC, white blood count; CRP, C-reactive protein; AST, aspartate aminotransferase; ALT, alanine aminotransferase; γGT, gamma-glutamyl transpeptidase; AP, alkaline phosphatase; IgG, immunoglobulin G. (DOCX 14 kb) [file 12893_2017_204_MOESM1_ESM.docx]

| Analyte name | **Results [Normal range]** |
| --- | --- |
| WBC count | 5.4 G/l [4-11] |
| Na^+^ | 139 mmol/l [136-144] |
| K^+^ | 4.1 mmol/l [3.6-4.6] |
| Ca^2+^ | 2.42 mmol/l [2.2-2.52] |
| CRP | **230 mg**/l [0-10] |
| AST | 25 U/l [11-42] |
| ALT | 24 U/l [9-42] |
| γGT | 51 U/l [9-35] |
| AP | 85 U/l [25.102] |
| Biluribin | <3 μmol/l [7-25] |
| Lipase | 24 U/l [13-60] |
| Amylase | 55 U/l [31.108] |
| Total Cholesterol | 5.4 mmol/l [<6.4] |
| Triglycerides | 2.08 mmol/l [<2] |
| IgG subclass IgG4 | 0.2 g/l [0-2] |

Add. File 1. Blood test results one day after the onset of the abdominal pain. WBC, white blood count ; CRP, C-reactive protein ; AST, [aspartate aminotransferase ; ALT,](http://www.webmd.com/digestive-disorders/aspartate-aminotransferase-ast) [alanine aminotransferase ; γGT, [gamma-glutamyl transpeptidase](https://medlineplus.gov/ency/article/003458.htm) ; AP, [alkaline phosphatase ; IgG, immunoglobuline G](http://www.healthline.com/health/alp)](http://www.webmd.com/digestive-disorders/alanine-aminotransferase-alt)

**Additional file 1**
